# Little evidence for sex or ovarian hormone influences on affective variability

**DOI:** 10.1038/s41598-021-00143-7

**Published:** 2021-10-22

**Authors:** Alexander Weigard, Amy M. Loviska, Adriene M. Beltz

**Affiliations:** Department of Psychology, The University of Michigan, 2227 East Hall 530 Church Street, Ann Arbor, MI 48109 USA

**Keywords:** Emotion, Human behaviour

## Abstract

Women were historically excluded from research participation partly due to the assumption that ovarian hormone fluctuations lead to variation, especially in emotion, that could not be experimentally controlled. Although challenged in principle and practice, relevant empirical data are limited by single measurement occasions. The current paper fills this knowledge gap using data from a 75-day intensive longitudinal study. Three indices of daily affective variability—volatility, emotional inertia, and cyclicity—were evaluated using Bayesian inferential methods in 142 men, naturally cycling women, and women using three different oral contraceptive formulations (that “stabilize” hormone fluctuations). Results provided more evidence for similarities between men and women—and between naturally cycling women and oral contraceptive users—than for differences. Even if differences exist, effects are likely small. Thus, there is little indication that ovarian hormones influence affective variability in women to a greater extent than the biopsychosocial factors that influence daily emotion in men.

## Introduction

Female animals and humans were excluded from biomedical, neurological, and social research for decades because cyclic fluctuations in ovarian hormones were assumed to induce variability that would undermine statistical inferences or experimental manipulations^1,2^. For decades, this assumption lacked empirical support. With funding and policy mandates, however, recent years have seen increased inclusion of females in scientific research^3–5^, and relevant data are beginning to emerge.

To-date, these data generally concern biological variability in rodents. Syntheses in several large-scale reviews and meta-analyses show that female mice are not more variable (and may even be less variable) than are males^6–8^. Of particular relevance is a meta-analysis on sex differences in physiological and trait variability that included over 300 studies and 6,000 data points in rats^9^. No sex differences in the variability of behavior, electrophysiology, histology, or neurochemistry measures were found, nor was variability impacted by the estrous cycle.

A recent study in mice^10^ went beyond these investigations of inter-individual variability (how females vary from each other) to focus on intra-individual variability (how an individual varies across assessments). This is an important distinction because estrous cycle fluctuations are known to affect intra-individual variability even if they are of little consequence for inter-individual variability. Continuous measurement of locomotion and body temperature across 12 days revealed that males were actually more variable across a day, but that females had a four-day cyclic pattern that coincided with their estrous cycle. Thus, there were sex differences in intra-individual variability, but the direction depended upon timescale (i.e., 24 versus 96 h).

Although studies about intra-individual variability in rodents are not directly applicable to human biology (let alone behavior), they are nonetheless important because there is scant evidence concerning sex differences in intra-individual variability of psychological traits in humans—even traits that fluctuate with time and are associated with ovarian hormones, such as emotion. Emotion varies meaningfully across moments, days, and the lifespan^11,12^. When assessed within and between days, intra-individual emotional variability can reflect individual differences and can even be considered a valid and reliable trait in its own right^11,13^. It is also related to personality, especially to neuroticism^11,14,15^, and to internalizing psychopathology in comprehensive meta-analyses^16–18^.

Unfortunately, there have been few investigations of sex differences in intra-individual affective variability, despite an extensive literature on inter-individual sex differences in emotion^19^. As noted by others^14^, most studies of intra-individual emotion variability simply fail to consider gender or attempt to statistically “control” for it^20–28^. This illustrates an interesting shift in the study of sex differences. Women were historically excluded due to concerns that variability would confound results, and now, women are included, but potential effects of sex are often deliberately ignored, posing problems for interpretation and effective prevention and intervention^3–5^.

Findings from the few studies that do consider affective variability are inconsistent. For instance, there is some indication that women have greater self-reported variability, especially for negative emotions, than do men^29,30^, but in other research, men and women do not differ in self reports of day-to-day distress^31^. These disparate findings are challenging to interpret because gender was not a focal variable in these aging samples (with declining ovarian function). Thus, as suggested by the unclear role of gender in a largescale meta-analysis on emotion variability, “more targeted research is needed”^17^ [p. 924].

Insight about sex differences in intra-individual variability in emotion, however, can be gleaned from studies of sex hormones. These studies show that sex hormone *levels* influence the brain and behavior, implying that emotion changes in concert with hormonal changes. Menstrual cycle studies (of natural rises and falls in endogenous ovarian hormones) and oral contraceptive (OC) studies (of self-administered exogenous ovarian hormones) are particularly insightful^32^. Menstrual cycle studies typically consider phases, including menstruation, follicular, and luteal. Although samples are often small and there are challenges in defining cycle phases^33^, findings generally show increased negative affect preceding and during menstruation when estrogen and progesterone are low^34–36^. This may be an indirect effect of physical premenstrual symptoms and menstruation discomfort and not a direct effect of ovarian hormones on emotion^37^.

OC studies typically include women following regimens of active hormone and placebo pills. Active pills vary in formulation and dose, with monophasic combined OCs containing a constant dose of estradiol and a progestin, and triphasic combined OCs containing variable doses of estradiol and/or a progestin. There is no clear evidence of exogenous hormone influences on emotion^38^. For instance, randomized trials suggest that OCs only impact emotion (both positively and negatively) for some women^39^, as a partial function of age, length of OC use, or pre-existing conditions. Within OC users and broadly consistent with findings from menstrual cycle studies, though, there is indication of worsening mood late in the active pill phase, extending into the placebo phase for some sub-samples of women^40–42^. Most studies also fail to consider effects of different pill formulations^32^—a critical limitation because work in other domains shows that especially the androgenicity of progestins matters for outcomes^43^.

Unique insight into ovarian hormone influences on affective variability comes from comparisons of naturally cycling (NC) women and OC users. A review of early studies is consistent with the intuition that OC users, especially users of monophasic pills, show less affective variability than NC women^44^, and recent research is generally consistent^35,42,45,46^. However, most studies concern inter-individual variability (despite the often rich repeated emotion assessments across phases), so it remains unclear whether OC users have reduced intra-individual variability in emotion.

### Current study

Despite emerging work on the importance of affective variability as a construct, questions remain about whether this construct displays systematic sex differences that may be associated with ovarian hormonal milieus. This study aimed to fill this knowledge gap by assessing evidence for sex-linked inter-individual differences in intra-individual variation in emotion. This was accomplished by using 75-day intensive longitudinal data from men, NC women, and women using three different types of OCs (two monophasic differing in progestin androgenicity and one triphasic). Intra-individual variability was operationalized by three indices of different emotion timescales—volatility (intra-individual standard deviation), emotional inertia (first order autoregression), and cyclic patterns (maximum significant autoregression that can reflect phases)—and compared across groups. Bayesian inferential methods were used for group comparisons, as the research question concerned quantification of the degree of evidence for group differences and the effect sizes of these differences.

## Methods

Data came from a parent study on sex hormones and behavior. Subsets concerning neuroticism and physical health^47^ as well as gender self-concept^48^ have been previously reported.

### Participants

Participants were 142 men (*n* = 30), NC women (*n* = 28), and OC users (*n* = 84), between 18 and 38 years old (*M* = 21.59; *SD* = 3.26) recruited from a university community and small U.S. city. Most were White/Caucasian (70%) and non-Hispanic (96%), with 23% Asian, 6% Black/African American, and < 1% multi-racial. Participants were not taking psychotropic or neuroendocrine medications, and they did not report physical health or reproductive conditions impacting hormone function (e.g., polycystic ovary syndrome). Women were never pregnant, and NC women reported regular menstrual cycles^43^. OC users were taking one of three pill formulations for at least three months^43^: monophasic containing ethinyl estradiol and the anti-androgenic progestin drospirenone (OCd; *n* = 22; e.g., Yaz, Nikki), monophasic containing ethinyl estradiol and the moderately androgenic progestin norethindrone acetate (OCna; *n* = 30; e.g., Microgestin, Loestrin), and triphasic containing ethinyl estradiol and three doses of the mildly androgenic progestin norgestimate (OCng; *n* = 32; e.g., Ortho-tri-cyclen, Trinessa).

Participants completed at least 80% of the 75 daily assessments; an additional 93 participants began the intensive longitudinal study, but 60 dropped out or were removed because they had response rates under 50%, and 31 completed with response rates between 51 and 79%; 2 participants were excluded for using an alternative OC formulation. The 80% cut-off was informed by research indicating that 20% missing time series data does not significantly impact inferences^49^, and empirical measure validation in this sample. Thus, 235 participants began the intensive longitudinal study, but 142 completed it and were included here.

Participant groups did not display statistically significant differences in age, *F*(2, 140) = 0.62, *p* = 0.542, or ethnicity, *χ*^*2*^(2) = 1.89, *p* = 0.389, but they did differ in race, *χ*^*2*^(6) = 12.97, *p* = 0.044, with more men endorsing an Asian identity (40%) than NC women (25%) or OC users (17%). One participant did not provide their age, four did not report ethnicity, and one did not endorse any racial identity. NC women and OC users also did not display a statistically significant difference in self-reported weight (*M* = 138.25, *SD* = 23.18), *t*(110) = 0.29, *p* = 0.772.

### Procedures

In an hour-long laboratory-based session, participants provided informed consent and completed questionnaires, including reports of their medication use and reproductive health history; OC users also presented their pill packet (or a picture thereof).

The following day, participants began the 75-day intensive longitudinal study. Every night, they completed an approximately 20-min online survey that assessed daily affect on any Internet-capable device using Qualtrics. They received a unique link to each day’s survey at 5:00PM through the Qualtrics system. Participants were asked to complete the survey after 8:00PM or before going to bed; the links expired the following day at noon. All research procedures were approved by the University of Michigan Institutional Review Board for Health Sciences and Behavioral Sciences (IRB-HSBS) and were conducted in accordance with the ethical standards outlined by the Declaration of Helsinki. All participants provided written informed consent.

Participants were compensated with either course credit or $15 for the laboratory session and up to $200 for the daily assessments. They received $1 for every assessment they completed. Compensation increased to $2 if they completed at least 80%, and they received a $50 bonus if they completed at least 90%. If their completion rate fell below 50% after 30 days, then they were withdrawn from the study. The average overall study completion rate (*N* = 235; including all who completed 100 days) was 71%, and the average completion rate for the sample reported here was 94%.

### Measures

In each daily assessment, participants completed the reliable and externally valid Positive and Negative Affect Schedule PANAS^50^, which has been widely used in investigations of inter-individual variability and employed in recent investigations of intra-individual variability^17^. Participants rated the extent to which they experienced 10 positive affect items (e.g., Happy, Proud) and 10 negative affect items (e.g., Irritable, Afraid) in the past 24 h on a 5-point scale (from 1 = “very slightly/not at all” to 5 = “extremely”). Daily composites were created by averaging across positive and negative affect items, respectively.

Three different indices of affective variability were generated for both positive and negative affect using R^51^; they each summarize variability across 75 days for each participant, but consider different timescales. First, *volatility* was operationalized by the intra-individual standard deviation (iSD). It is the extent to which each individual’s affect varied from their own mean. It is not locked to a timescale, and so is not affected by missing data and reflects a composite of emotional peaks and valleys. It is also the most common measure of affective intra-individual variability^17^. Second, *emotional inertia* was operationalized by autocorrelation, (i.e., autoregressive coefficients for a lag of *t*–1); missing values were imputed using time series-based linear interpolation. This index reflects the extent to which today’s affect predicts tomorrow’s affect, or the daily carryover or persistence of emotion, and has been informative in recent investigations of affective variability^17^. Third, *cyclicity* was operationalized by the longest significant lag from *t*–1 through *t*–7 (i.e., the extent to which today’s affect predicts affect 7 days from now) again using linear interpolation to account for missing data. This novel index reflects the length of cyclic temporal patterns in daily emotion. For instance, a significant lag 5 could indicate a weekday/weekend cycle, which has been detected in previous work^52^.

### Statistical analyses

Group comparisons were conducted for each index of positive and negative affective variability. Sex differences in affective variability were examined by comparing men (reference group) and NC women (focusing on endogenous ovarian hormones), and by comparing men to all women (both NC and OC, reflecting the general population, which is a mix of women who do, and do not, use hormonal contraceptives). Although the former comparison provides a controlled test of the hypothesis for naturally-occurring sex differences in affect variability, the latter has practical implications for samples recruited from the general population. Next, differences between NC women (reference group) and OC users (considering OCd, OCna, and OCng users separately) were examined to assess effects of exogenous ovarian hormones on women’s affective variability.

For all group comparisons, Bayesian and frequentist *t*-tests were conducted using JASP Version 0.8.6^53,54^. Bayesian *t*-tests involve the comparison of prior and posterior probability distributions for a standard effect size parameter, δ. The prior distribution represents beliefs about the value of δ before seeing the data. As there is little information that can be used to form strong priors for δ here, the JASP default prior was used: a zero-centered Cauchy distribution with a scale of 0.707. This prior represents relatively weak beliefs about possible values of δ, holding that the effect is most likely to be small, while not ruling out the possibility of large effects (e.g., δ > 1) in either direction. The posterior distribution represents updated beliefs about the value of δ after “seeing” the data. The focus here is on the median of the posterior distribution, which indicates the most likely δ value, and on the 95% posterior credible interval (CI), which indicates the range in which δ has a 95% chance of falling.

Posteriors are only interpretable if the effect in question exists (i.e., δ ≠ 0). To quantify evidence that the effect exists, the difference between the density of the prior and posterior distributions at 0 is used to compute Bayes factors, which represent how beliefs about the null hypothesis (δ = 0) and alternative hypothesis (δ ≠ 0) change given the data. Bayes factors for the null (BF_01_) and alternative (BF_10_) hypotheses are interpreted as odds ratios; for example, a BF_01_ of 3 (identical to a BF_10_ = 0.33) indicates the data are three times more likely under the null than under the alternative hypothesis. Although Bayes factors are interpreted on a continuous scale, conventions for categorizing degrees of evidence have also been proposed: Values between 1 and 3 provide “anecdotal” evidence for a hypothesis, values between 3 and 10 provide “moderate” evidence, and values > 10 provide “strong” evidence^53^.

## Results

Results are presented in two parts. First, interrelations among the affective variability measures are reported and illustrated via plots of daily data from example participants. Second, group comparison results from Bayesian *t*-tests are presented. Bayesian analyses were of primary interest, but results from frequentist *t*-tests, which generally aligned with Bayesian results, are reported in the Supplemental Materials for completeness (Supplemental Table 1).

### Descriptive statistics

Correlations among affective variability indices are shown in Table 1; descriptive statistics are reported in Supplemental Table 2. Correlations had an average magnitude of 0.22, reflecting limited overlap among emotional volatility, inertia, and cyclicity, although correlations did range from negligible to large. Individual scores on each index are shown in scatterplots in Supplemental Fig. 1 by group. To the naked eye, the means and ranges appear similar across groups, especially for volatility and inertia.

**Table 1:** Bayesian estimates of correlations among measures of daily positive and negative affective variability, including volatility (intra-individual standard deviation), inertia (strength of first order autoregressions), and cyclicity (longest order from 0 through 7 with a significant autoregression).

	Pos. Volatility	Neg. Volatility	Pos. Inertia	Neg. Inertia	Pos. Cyclicity
Pos. Volatility	–				
	–				
	–				
Neg. Volatility	**0.40**	–			
	*0.53*	–			
	*0.25*	–			
Pos. Inertia	**0.06**	− **0.15**	–		
	*0.22*	*0.02*	–		
	− *0.11*	− *0.30*	–		
Neg. Inertia	− **0.06**	**0.32**	**0.29**	–	
	*0.11*	*0.45*	*0.43*	–	
	− *0.22*	*0.16*	*0.13*	–	
Pos. Cyclicity	**0.00**	− **0.17**	**0.52**	**0.14**	–
	*0.16*	− *0.01*	*0.63*	*0.29*	–
	− *0.17*	− *0.33*	*0.38*	− *0.03*	–
Neg. Cyclicity	− **0.18**	**0.23**	**0.08**	**0.48**	**0.18**
	− *0.02*	*0.37*	*0.24*	*0.60*	*0.33*
	− *0.34*	*0.06*	− *0.09*	*0.34*	*0.01*

Measures were calculated separately for positive (Pos.) and negative (Neg.) affect. Numbers in bold indicate the median of the posterior distribution for Pearson’s *r*, which is the most likely *r* value, while numbers in italics indicate the upper and lower bounds of the 95% posterior credible interval, which is the range in which there is a 0.95 probability that the *r* value falls.

**Figure 1:**
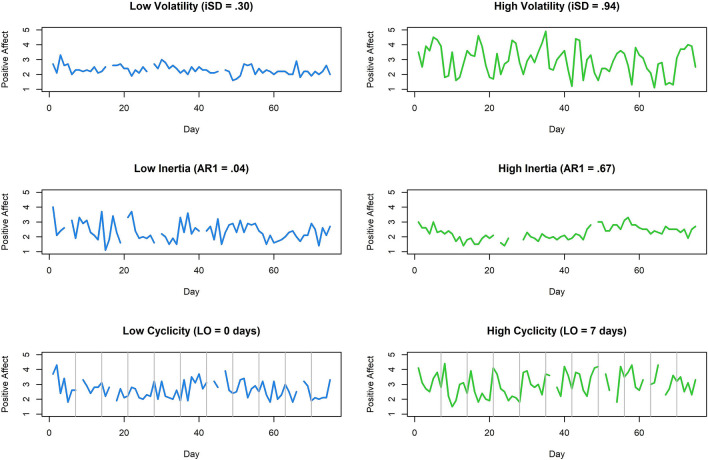
Example 75-day time series plots for participants who had relatively low (< 25th percentile) and relatively high (> 75th percentile) values on positive affective variability indices: Volatility**,** or intra-individual standard deviation (iSD); Inertia, or the strength of the first order autoregressive relation (AR1); and Cyclicity, or the longest order (LO) between 0 and 7 days for which there was a statistically significant autoregressive relation. Gray lines in plots of Cyclicity indicate every 7th day.

To illustrate individual differences in indices of affective variability, Fig. 1 depicts the positive affect time series from example participants who had relatively low (< 25th percentile) and relatively high (> 75th percentile) values on each index. The participant with high volatility displays positive affect scores with repeated high amplitudes that drop off compared to the participant with low volatility, whose scores seem to hover around an imaginary mean line. Individual differences in emotional inertia are comparatively subtle. Although the participant with high inertia has scores that vary over a small range, the primary distinction is that there are only small jumps from day-to-day. Interestingly, those jumps lead to progressive shifts in positive affect across days (e.g., decline through day 15 followed by increase until days 50–60). There is little predictability, however, in positive affect scores for the participant with low emotional inertia. The participant with high cyclicity displays a clear pattern of increases followed by decreases within most 7-day periods (denoted by gray lines). The participant with a low, 0-day cycle displays no such patterns.

### Group differences

**Figure 2:**
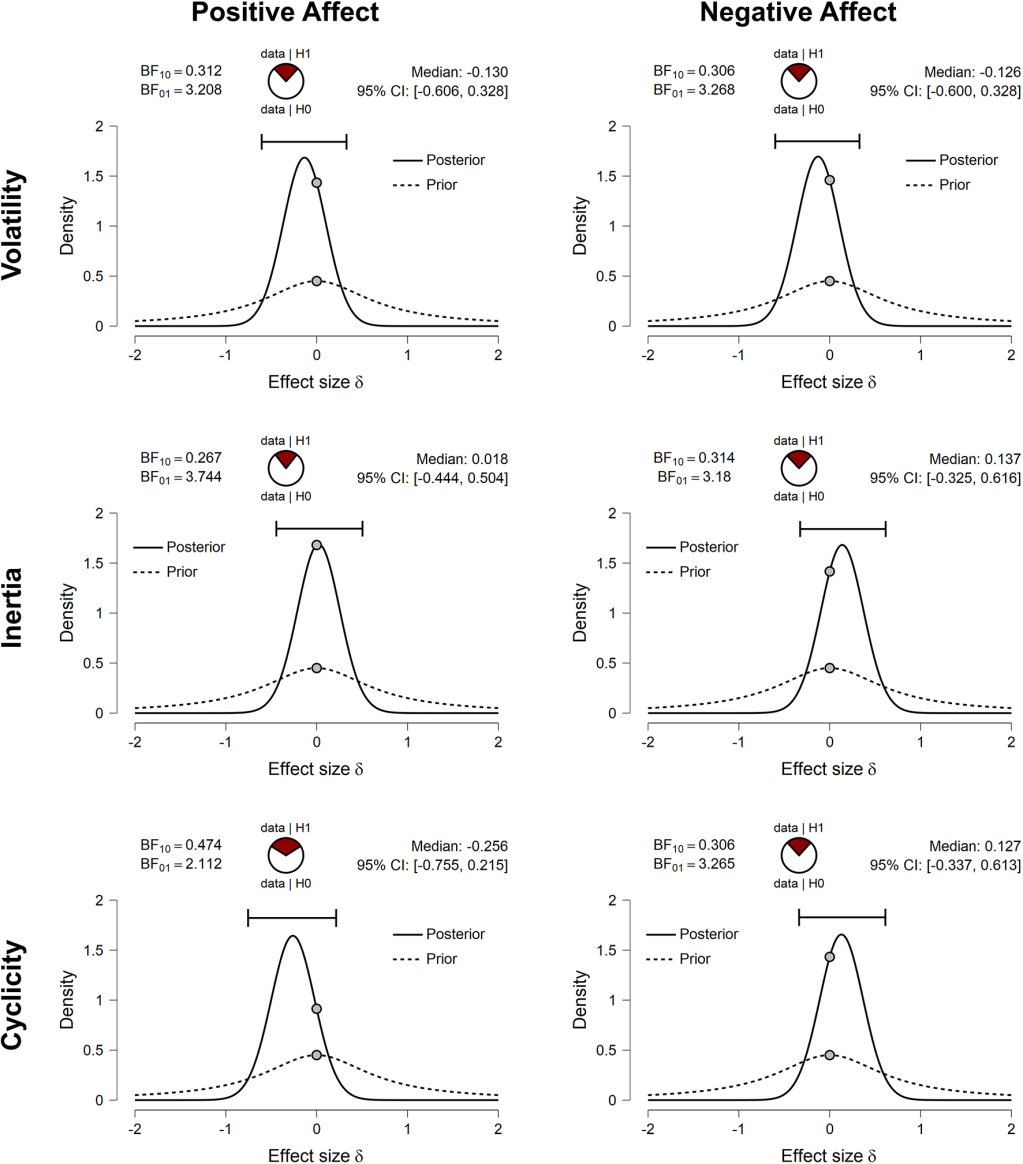
JASP^54^ graphical output for the comparison between men and naturally cycling (NC) women on each positive and negative affective variability index using Bayesian *t*-tests. Density plots indicate the prior probability distribution (dashed lines) and posterior probability distribution (solid lines) of the effect size (δ) for each test, with gray dots indicating the prior and posterior probability of δ = 0 (the point null hypothesis reflecting no sex difference) and the black horizontal error bar indicating the 95% credible interval (CI) of the posterior distribution (i.e., likely size of a sex difference if one exists). Bayes factors for the alternative hypothesis (δ ≠ 0; BF_10_) and the point null hypothesis (δ = 0; BF_01_), proportion wheels representing the strength of evidence each Bayes factor provides^53^, and the values of the posterior median and CI are all reported above each density plot. Note that men were the reference group, and therefore, positive values of δ indicate that the index is greater in NC women, while negative values of δ indicate that the index is lower in NC women.

**Figure 3:**
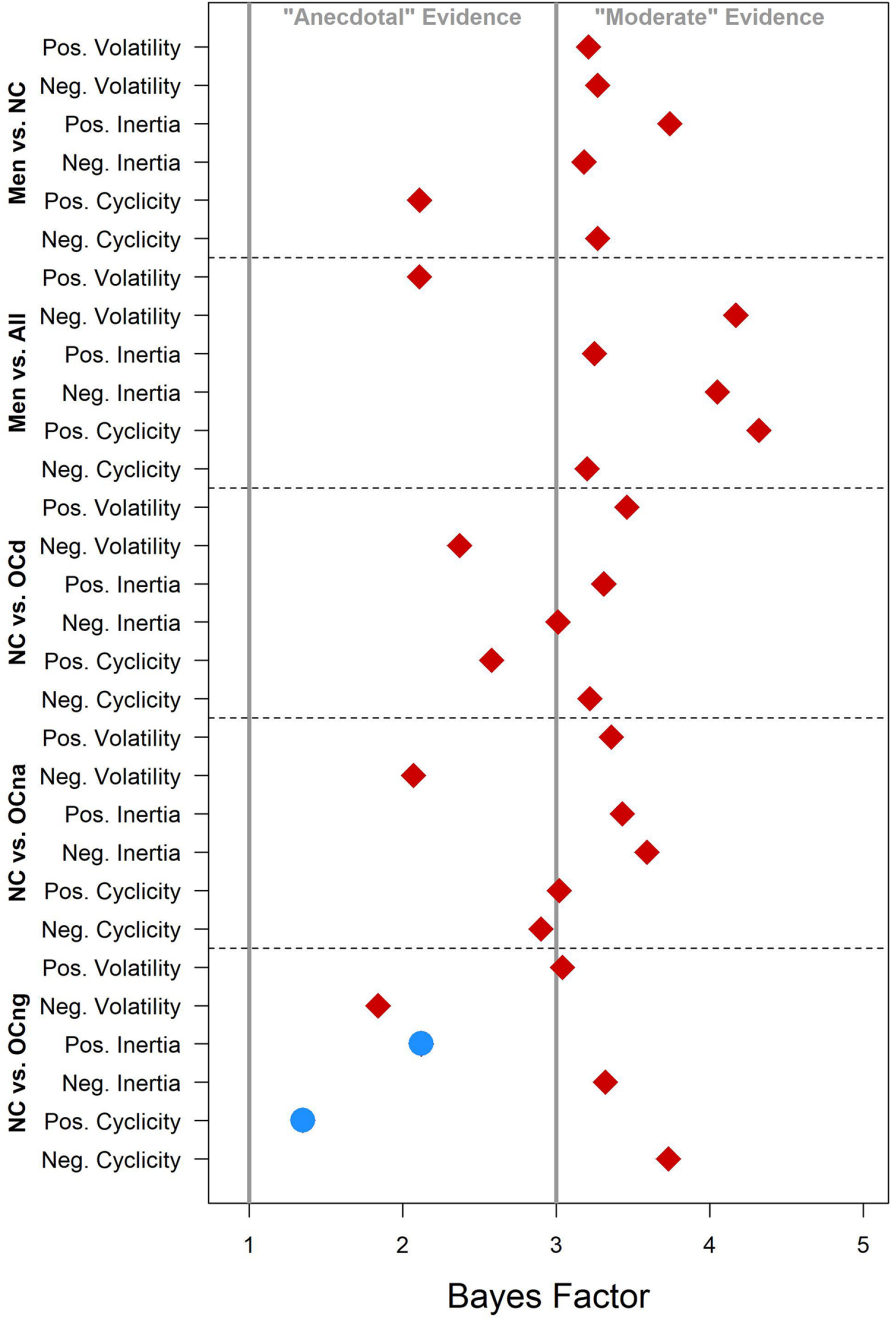
Bayes factors for group comparisons of affective variability. Red diamonds represent evidence in favor of the null hypothesis (BF_01_), and blue circles represent evidence in favor of the alternative hypothesis (BF_10_). Gray lines separate the ranges in which Bayes factors are typically interpreted as providing only “anecdotal” evidence (1 < BF < 3) versus “moderate” evidence (3 < BF < 10) for a hypothesis^53^. NC, naturally cycling women; OCd, OC users of pills with ethinyl estradiol and drospirenone; OCna, OC users of pills with ethinyl estradiol and norethindrone acetate; OCng, OC users of pills with ethinyl estradiol and norgestimate.

**Figure 4:**
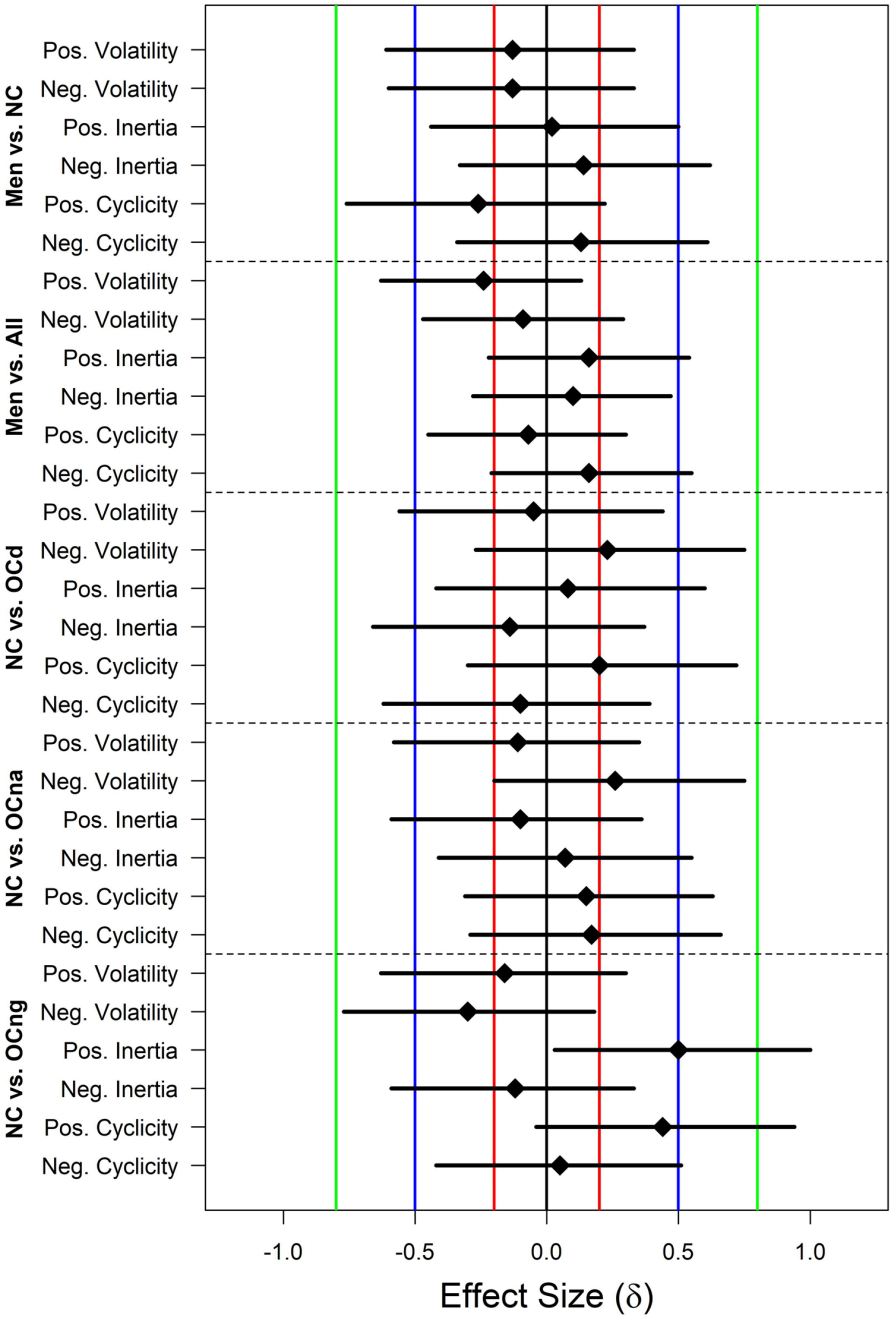
Graphical summary of the medians (diamonds) and credible intervals (CIs; solid black lines) of posterior distributions for effect size (δ) from all Bayesian *t*-tests of group differences in indices of affective variability. The dark gray line in the center indicates an effect size of 0, while the colored lines indicate δ values commonly labeled as “small” (red: δ =|0.20|), “medium” (blue: δ =|0.50|), and “large” (green: δ =|0.80|). NC, naturally cycling women; OCd, OC users of pills with ethinyl estradiol and drospirenone; OCna, OC users of pills with ethinyl estradiol and norethindrone acetate; OCng, OC users of pills with ethinyl estradiol and norgestimate.

Comparisons between men and NC women provided the primary test of sex differences in affective variability, and detailed Bayesian *t*-test results for these comparisons are shown in Fig. 2. The density plots illustrate the weak prior probability distribution for effect size δ (dashed line), the posterior probability distribution (solid line), and the difference in the density of these distributions at δ = 0 (gray dots), which is used to estimate the Bayes factor. For comparisons of all affective variability indices, the point null hypothesis (δ = 0, or no sex difference) was more likely after consideration of the data. This is reflected in the BF_01_ values, displayed for men versus NC comparisons in Fig. 2 and for all comparisons in Fig. 3, which indicate that the null is between 2.1 and 3.7 times more likely than the alternative hypothesis (δ ≠ 0, or a sex difference). Furthermore, the posterior density plots and CIs, displayed for men versus NCs in Fig. 2 and for all comparisons in Fig. 4 (with reference lines indicating effect sizes typically considered “small”, δ =|0.20|, “medium”, δ =|0.50|, and “large” δ =|0.80|), show that, in the event that the effect size is not *exactly* 0, the most likely effect sizes are very small. Hence, these group differences are unlikely to be of major consequence, even if they do exist.

Comparisons between men and all women revealed moderate evidence for the null in all cases except for positive volatility, in which evidence was only anecdotal (BF_01_ = 2.11). The range of effect sizes for positive volatility, however, was modest and mostly negative (Fig. 4), indicating that if a difference exists, then men may actually be slightly more variable than women. Posterior medians for the remaining comparisons between men and all women were close to 0 and CIs rarely exceeded small effects.

Similar patterns held for comparisons between NC women and the OCd and OCna users, visualized in Figs. 3 and 4: There was more evidence for the null hypothesis than for group differences, and the most likely effect sizes ranged from small to near-0 with CIs showing only slight overlap with medium effect sizes. Evidence for some comparisons between NC women and the OCng group, however, was mixed, as there was anecdotal evidence for differences in positive inertia (BF_10_ = 2.12) and positive cyclicity (BF_10_ = 1.35). The posterior medians and CIs for these comparisons indicated that, if such differences exist, women using triphasic pills with norgestimate have greater inertia and cyclicity, suggesting reduced variability.

## Discussion

Implicit assumptions about how fluctuations in ovarian hormones relate to variability in emotion have contributed to the exclusion of women from research, and thus, have stymied understanding of female behavior. Although recent animal research provides little support for sex differences in trait variability, conclusions concerning human emotion—despite increasing evidence of affective variability as a marker of individual differences—are unclear. The goal of the present study was to directly address this knowledge gap in a sample of men and women with varying ovarian hormonal milieus using three indices of affective variability calculated from 75 daily assessments per person: (1) *volatility*; (2) *emotional inertia*; and (3) *cyclicity*.

Results provide little evidence for sex differences, and thus, empirically undermine the notion that excluding females from research studies improves inferences about affect or emotion. Specifically, evidence from Bayes factors for the lack of sex differences was an average of 3.3 times greater than evidence for the existence of sex differences. Moreover, even if sex differences exist, their most likely effect sizes (δ posterior medians) were very small and suggested that, if anything, men had more variability in positive affect than women.

These findings converge with early work on the lack of sex differences in rodent trait variability, suggesting that ovarian hormones either do not matter for affective variability, or that “male[s] feature their own sex-specific variability”^8^ [p. 3]. Thus, men and women may have similar levels of affective variability, but the mechanisms or biopsychosocial processes that underlie this variability may systematically differ between the sexes. One contributing mechanism could be ovarian hormones, which influence emotion in *some* women^38,42^, and contributing mechanisms for *some* men could be testosterone or personality traits linked to dominance or aggression^55^. As effects are unlikely to be homogeneous even within the sexes, future work should broadly consider sources of individual differences in affective variability.

Results also provide little evidence that affective variability is altered by systematic shifts in ovarian hormones. In comparisons between NC women and women using OCs with different formulations (i.e., varying in progestin androgenicity) and doses (i.e., monophasic versus triphasic administrations), evidence for the lack of group differences was an average of 2.7 times greater than evidence for differences. Most effect size estimates for differences were also very small to small, consistent with conclusions from early work and—once again—suggesting that ovarian hormones have only a small influence on emotion that is often superseded by other biopsychosocial influences^56^. The comparison between NC women and women using triphasic OCs with norgestimate (OCng), however, ran counter to this trend. Specifically, there was modest evidence that OC users may have greater positive emotional inertia and cyclicity. This finding must be replicated, especially because past work provides little interpretative context, as it largely focused on inter-individual variability and negative affect, and did not consider OC formulations and doses^38,44^.

Together, results provide little evidence for ovarian hormone-related inter-individual differences in intra-individual variation in emotion across 75 days. Past research has documented many influences on both sex hormones, ranging from biological cycles to daily experiences, including diet, sleep, social interactions, and physical exertion^33^, as well as direct links between ovarian hormone levels and emotions^34–36^. This study does not speak to those direct associations. Instead, it speaks to overall variation in emotion—whatever its origins—and whether it systematically differs in men and women or across women with varying hormonal milieus. Indeed, if ovarian hormone influences on emotion variation were so great in the everyday lives of women—great enough to exclude women from scientific research participation, for instance—then women would be expected to show clear differences in overall emotion variation from men or from each other according to their hormone profiles (e.g., having a natural menstrual cycle versus using OCs with varying pharmacokinetic properties). This study, however, does not support such conclusions.

### Study considerations

There are five key features of the study that warrant additional comment. The first is linked to sample characteristics and concerns generalizing study findings. Participants were from a Western, industrialized nation, and the majority were young, White, non-Hispanic, and affiliated with a university. There were also disproportionately more Asian men than Asian NC women or OC users in the sample. Given evidence for varying sex hormone processes and the ways in which they relate to lifestyle factors across development, ethnoracial identities, and cultures^57,58^, it is imperative that future work recruit individuals of diverse ages and identities, and from international, non-industrialized communities.

The second concerns emotion assessment. Although widely-used indicators of positive and negative affect were employed^50,59^, corroborating measures of emotion were not available from the parent study, and findings may differ for clinical indicators of mood, particularly depression, which shows a sex difference and link to hormonal contraceptive use^60,61^. Similarly, variability in *daily* emotion was operationalized via three indicators, but measures from different timescales (e.g., hourly) or different operationalizations (e.g., from spectral or network analyses) could provide additional insight^18,62,63^. Finally, it is unclear whether time of day was related to emotion reports, especially because participants could complete daily surveys up until noon the next day, but there is no reason to think that men and women responded at systematically different times. The daily indices used here are likely reasonable: They align with a trait-like conceptualization of affective variability^11^, have relatively low inter-relations^14^, and yet, produced consistent evidence.

The third concerns ovarian hormones. It is important to emphasize that findings do not have implications for the understanding of direct links between ovarian hormones and emotion (e.g., whether high levels of progesterone during the luteal phase of the menstrual cycle predict negative affect), although an exciting opportunity for future research concerns delineating these and other lifestyle factors that influence emotion variability in men and women. The study focused on group differences in trait-like affective variability (using repeated within-person assessments across two cycles)—not on comparing cycle or pill phases. Phase determination without daily hormone assessments, which are unfortunately not available in this sample, and without granular knowledge of OC use (e.g., number and timing of placebo pill days) is a process riddled with error^33^.

The fourth concerns the relatively small group sizes. In frequentist hypothesis testing, they were only large enough to reliably detect moderate-to-large effects at conventional thresholds, which is why *p*-values were provided only for reference in Supplemental Materials. However, Bayesian hypothesis testing produced continuous estimates of evidence for and against effects in these small samples by directly quantifying uncertainty about their possible presence and size. Hence, Bayesian methods leveraged the information provided by the small groups, which are unprecedented in terms of within-person measurement for women with varying ovarian hormone profiles, in order to draw converging conclusions: Sex and ovarian hormone-related differences in affective variability are unlikely to be large enough to be practically meaningful.

The fifth concerns the use of Bayesian inferential methods. The focus was on evaluating evidence for each effect compared to a point null hypothesis using Bayes factors, and then investigating the range of possible effect sizes with posterior medians and CIs. Although the latter is only applicable if the null hypothesis is false^53^, it is nonetheless valuable to consider CIs because evidence in favor of the null was not overwhelming (i.e., BF_01_ was generally between 2 and 4), and because the point null hypothesis of δ = 0 is often implausible^64,65^.

## Conclusions

The goal of this study was to fill the significant knowledge gap concerning sex differences in affective variability and its ovarian hormone links. Using 75 daily assessments of emotion (indexed by three different timescales) in men, NC women, and women using three different types of OCs (that are thought to diminish ovarian hormone fluctuations), Bayesian inferential methods generally found evidence for group similarities (i.e., the null hypothesis) to be roughly three times greater than evidence for differences (i.e., the alternative hypothesis), and indicated that effect sizes, even if differences do exist, are likely to be very small. Thus, daily emotion fluctuates to similar extents in men and women: There may be sex differences in the factors that influence emotion, such as ovarian hormones, but those factors do not ultimately produce different outcomes with respect to affective variability. Biomedical, neurological, and social scientists are encouraged to adjust their conceptual and statistical priors accordingly.

## Supplementary Information

Supplementary Information.

## Data Availability

The data generated and analyzed during the current study are available from the corresponding author upon reasonable request.
