# Supplementary material for: Little evidence for sex or ovarian hormone influences on affective variability
Source: Sci Rep. 2021 Oct 22;11:20925. doi: 10.1038/s41598-021-00143-7 (PMC8536752; doi:10.1038/s41598-021-00143-7)

**Supplemental Materials for**:

**Little evidence for sex or ovarian hormone influences on affective variability**

Alexander Weigard, Amy Loviska, and Adriene M. Beltz

*The University of Michigan*

**Results of Frequentist Tests**

All relevant test statistics from frequentist and Bayesian *t*-tests are reported in detail in Supplemental Table 1. Frequentist tests of all possible sex differences and contraceptive-related differences revealed only one instance in which the null hypothesis could be rejected at the conventional alpha level (.05); this result suggested that women in the OCng group displayed greater positive inertia than the those in the NC group *t*(58)=2.25, *p*=0.028. However, this result does not survive even a relatively liberal correction for multiple comparisons (FDR<.05; families of tests defined by type of comparison), suggesting that it may be a spurious finding. Therefore, frequentist analyses do not provide any strong indication that the default null hypotheses – the hypotheses that affect variability is unaffected by biological sex and/or by exogenous sex hormones in women – should be rejected.

**Supplemental Table 1**. Results from Bayesian and frequentist *t*-tests for all effects of interest. Bayesian *t*-test results include: 1) the median of the posterior distribution of the effect size (Post. Med. δ), representing the most likely value of δ, 2) 95% credible intervals, representing the lower (2.5% CI δ) and upper (97.5% CI δ) bounds of the range that has a .95 probability of containing δ, and 3) Bayes factors for the alternative hypothesis (δ ≠ 0; BF_10_) and for the point null hypothesis (δ = 0; BF_01_). Frequentist *p*-values reported are uncorrected for multiple comparisons (none survived later correction, as noted in the text). NC = naturally cycling women; OCd = drospirenone; OCna = norethindrone acetate; OCng = norgestimate

| Test | Variability Measure | Post. Med. δ | 2.5% CI δ | 97.5% CI δ | BF_10_ | BF_01_ | *t* | *p* |
| --- | --- | --- | --- | --- | --- | --- | --- | --- |
| Men vs. NC | Pos. Volatility | -0.13 | -0.61 | 0.33 | 0.31 | 3.21 | -0.61 | 0.542 |
|  | Neg. Volatility | -0.13 | -0.60 | 0.33 | 0.31 | 3.27 | -0.58 | 0.567 |
|  | Pos. Inertia | 0.02 | -0.44 | 0.50 | 0.27 | 3.74 | 0.09 | 0.929 |
|  | Neg. Inertia | 0.14 | -0.33 | 0.62 | 0.31 | 3.18 | 0.63 | 0.531 |
|  | Pos. Cyclicity | -0.26 | -0.76 | 0.22 | 0.47 | 2.11 | -1.18 | 0.245 |
|  | Neg. Cyclicity | 0.13 | -0.34 | 0.61 | 0.31 | 3.27 | 0.58 | 0.565 |
| Men vs. All Women | Pos. Volatility | -0.24 | -0.63 | 0.13 | 0.47 | 2.11 | -1.34 | 0.184 |
|  | Neg. Volatility | -0.09 | -0.47 | 0.29 | 0.24 | 4.17 | -0.49 | 0.628 |
|  | Pos. Inertia | 0.16 | -0.22 | 0.54 | 0.31 | 3.25 | 0.90 | 0.371 |
|  | Neg. Inertia | 0.10 | -0.28 | 0.47 | 0.25 | 4.05 | 0.55 | 0.582 |
|  | Pos. Cyclicity | -0.07 | -0.45 | 0.30 | 0.23 | 4.32 | -0.40 | 0.692 |
|  | Neg. Cyclicity | 0.16 | -0.21 | 0.55 | 0.31 | 3.20 | 0.92 | 0.361 |
| NC vs. OCd | Pos. Volatility | -0.05 | -0.56 | 0.44 | 0.29 | 3.46 | -0.21 | 0.836 |
|  | Neg. Volatility | 0.23 | -0.27 | 0.75 | 0.42 | 2.37 | 0.98 | 0.331 |
|  | Pos. Inertia | 0.08 | -0.42 | 0.60 | 0.30 | 3.31 | 0.39 | 0.699 |
|  | Neg. Inertia | -0.14 | -0.66 | 0.37 | 0.33 | 3.01 | -0.62 | 0.539 |
|  | Pos. Cyclicity | 0.20 | -0.30 | 0.72 | 0.39 | 2.58 | 0.87 | 0.389 |
|  | Neg. Cyclicity | -0.10 | -0.62 | 0.39 | 0.31 | 3.22 | -0.46 | 0.645 |
| NC vs. OCna | Pos. Volatility | -0.11 | -0.58 | 0.35 | 0.30 | 3.36 | -0.52 | 0.607 |
|  | Neg. Volatility | 0.26 | -0.20 | 0.75 | 0.48 | 2.07 | 1.20 | 0.237 |
|  | Pos. Inertia | -0.10 | -0.59 | 0.36 | 0.29 | 3.43 | -0.47 | 0.641 |
|  | Neg. Inertia | 0.07 | -0.41 | 0.55 | 0.28 | 3.59 | 0.33 | 0.746 |
|  | Pos. Cyclicity | 0.15 | -0.31 | 0.63 | 0.33 | 3.02 | 0.72 | 0.475 |
|  | Neg. Cyclicity | 0.17 | -0.29 | 0.66 | 0.35 | 2.90 | 0.79 | 0.434 |
| NC vs. OCng | Pos. Volatility | -0.16 | -0.63 | 0.30 | 0.33 | 3.04 | -0.73 | 0.469 |
|  | Neg. Volatility | -0.30 | -0.77 | 0.18 | 0.54 | 1.84 | -1.32 | 0.192 |
|  | Pos. Inertia | 0.50 | 0.03 | 1.00 | 2.12 | 0.47 | 2.25 | 0.028 |
|  | Neg. Inertia | -0.12 | -0.59 | 0.33 | 0.30 | 3.32 | -0.57 | 0.570 |
|  | Pos. Cyclicity | 0.44 | -0.04 | 0.94 | 1.35 | 0.74 | 1.99 | 0.051 |
|  | Neg. Cyclicity | 0.05 | -0.42 | 0.51 | 0.27 | 3.73 | 0.22 | 0.826 |

**Supplemental Table 2**. Descriptive statistics of all positive and negative affect variability measures in each group, including the mean, standard deviation (Std. Dev.), minimum value (Min.) and maximum value (Max.).

|  |  | **Men** | **NC** | **OCd** | **OCna** | **OCng** |
| --- | --- | --- | --- | --- | --- | --- |
| Positive Volatility | **Mean** | 0.53 | 0.50 | 0.50 | 0.49 | 0.48 |
|  | **Std. Dev.** | 0.20 | 0.11 | 0.13 | 0.13 | 0.12 |
|  | **Min.** | 0.18 | 0.27 | 0.24 | 0.26 | 0.30 |
|  | **Max.** | 0.99 | 0.74 | 0.82 | 0.76 | 0.74 |
| Negative Volatility | **Mean** | 0.42 | 0.39 | 0.44 | 0.45 | 0.34 |
|  | **Std. Dev.** | 0.19 | 0.18 | 0.19 | 0.19 | 0.16 |
|  | **Min.** | 0.12 | 0.10 | 0.19 | 0.18 | 0.08 |
|  | **Max.** | 0.98 | 0.81 | 0.96 | 0.94 | 0.81 |
| Positive Inertia | **Mean** | 0.32 | 0.33 | 0.34 | 0.31 | 0.44 |
|  | **Std. Dev.** | 0.20 | 0.17 | 0.12 | 0.16 | 0.20 |
|  | **Min.** | -0.12 | 0.03 | 0.12 | -0.02 | 0.09 |
|  | **Max.** | 0.75 | 0.67 | 0.52 | 0.56 | 0.78 |
| Negative Inertia | **Mean** | 0.32 | 0.35 | 0.32 | 0.37 | 0.32 |
|  | **Std. Dev.** | 0.17 | 0.19 | 0.19 | 0.17 | 0.17 |
|  | **Min.** | 0.00 | -0.14 | 0.02 | 0.04 | -0.02 |
|  | **Max.** | 0.75 | 0.72 | 0.82 | 0.72 | 0.72 |
| Positive Cyclicity | **Mean** | 3.03 | 2.25 | 2.82 | 2.67 | 3.50 |
|  | **Std. Dev.** | 2.83 | 2.17 | 2.44 | 2.23 | 2.63 |
|  | **Min.** | 0.00 | 0.00 | 0.00 | 0.00 | 0.00 |
|  | **Max.** | 7.00 | 7.00 | 7.00 | 7.00 | 7.00 |
| Negative Cyclicity | **Mean** | 2.50 | 2.86 | 2.55 | 3.37 | 3.00 |
|  | **Std. Dev.** | 2.46 | 2.22 | 2.52 | 2.66 | 2.71 |
|  | **Min.** | 0.00 | 0.00 | 0.00 | 0.00 | 0.00 |
|  | **Max.** | 7.00 | 7.00 | 7.00 | 7.00 | 7.00 |

**Supplemental Figure 1**. Scatterplots of individual values (colored dots) by group, with means (black bars) for each index of positive and negative affective variability. Men are in blue, naturally cycling (NC) women are dark red, and oral contraceptive (OC) users grouped by pill formulation are in lighter shades of red: OCd, OC users of pills with ethinyl estradiol and drospirenone; OCna: OC users of pills with ethinyl estradiol and norethindrone acetate; OCng: OC users of pills with ethinyl estradiol and norgestimate.


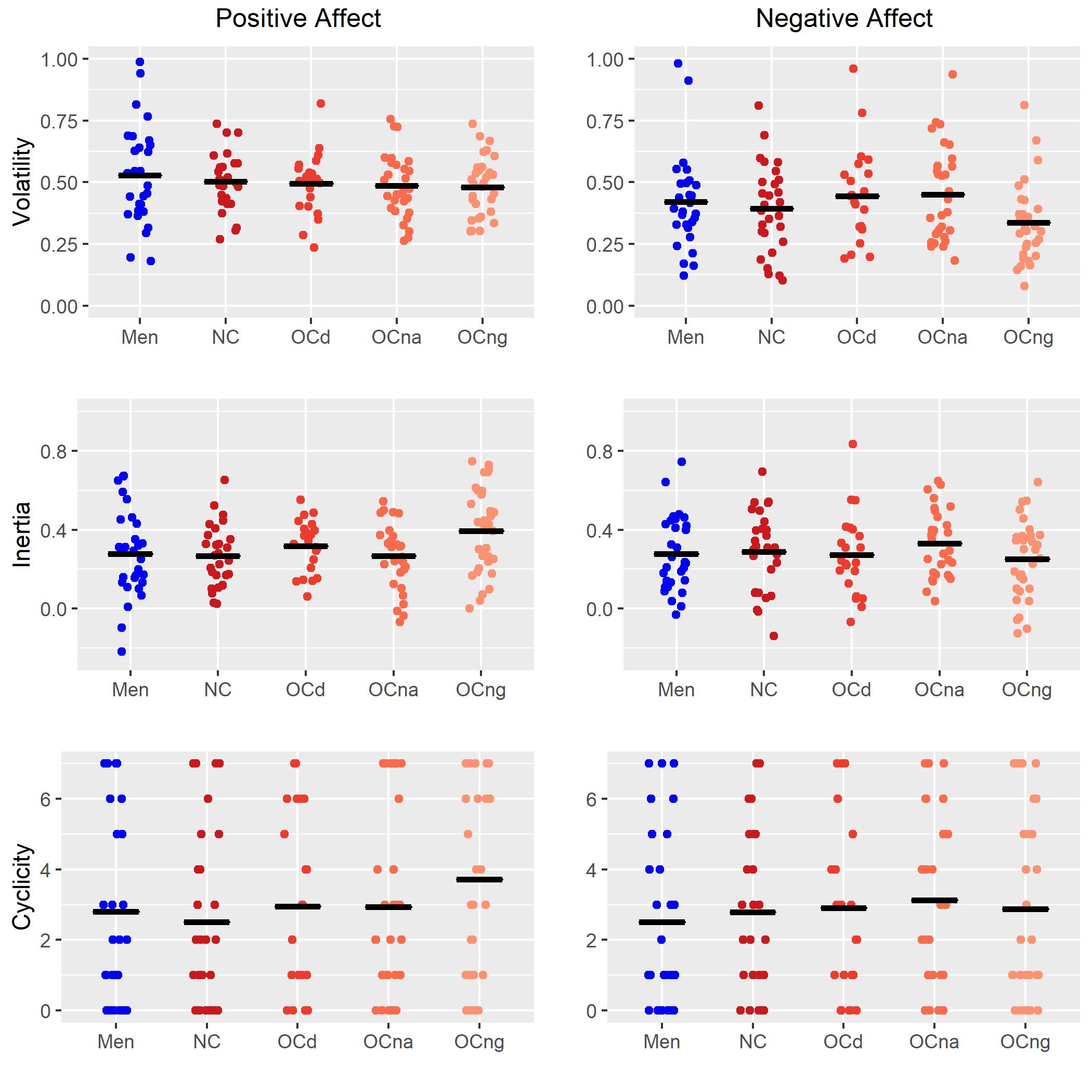

Supplement: Supplementary file 1 — Supplementary Information. [file 41598_2021_143_MOESM1_ESM.docx]
